# Barriers to comprehensive fertility counseling among persons living with HIV/AIDS in Ghana

**DOI:** 10.3389/frph.2026.1914739

**Published:** 2026-10-16

**Authors:** Priscilla Asantewaa Boadi, Victor Luckyboy Dzramado

**Affiliations:** 1 Department of Nursing, School of Nursing and Midwifery, College of Health Sciences, Kwame Nkrumah University of Science and Technology, Kumasi, Ghana; 2 Department of Health Information Management, University of Cape Coast, Cape Coast, Ghana; 3 Cape Coast Teaching Hospital, Cape Coast, Ghana

**Keywords:** antiretroviral therapy, barriers, fertility counseling, Ghana, people living with HIV, qualitative research, reproductive health, safer conception

## Abstract

**Introduction:**

Persons living with HIV/AIDS (PLWHA) in Ghana increasingly express the wish to have children, yet little is known about whether routine HIV care delivers fertility counseling that extends beyond prevention of mother-to-child transmission to the safer conception, individualized planning, and onward referral that comprehensive counseling requires. This study explored the barriers to comprehensive fertility counseling experienced by PLWHA receiving antiretroviral therapy at a district hospital in the Ashanti Region of Ghana.

**Methods:**

A qualitative interpretive phenomenological design was used. Twelve purposively selected adults living with HIV, comprising nine women and three men aged 26–49 years, attended the antiretroviral therapy clinic at St. Michael's Hospital, Pramso, and took part in in-depth semi-structured interviews between August and October 2024. Interviews were audio-recorded, transcribed verbatim, and analyzed manually through an inductive interpretive phenomenological process that moved from meaning units to situated interpretation and then to essential themes. Trustworthiness was supported through reflexive journaling, documented positionality, participant validation of preliminary interpretations, and an audit trail.

**Results:**

Three essential themes captured how healthcare experiences shaped fertility counseling and decision-making, namely quality of reproductive health counseling and education, healthcare provider attitudes and support, and access to comprehensive reproductive health services. Participants valued prevention of mother-to-child transmission education and the relational warmth of providers, yet described counseling that was generic rather than tailored to their circumstances and that seldom addressed protection of an HIV-negative partner during conception. No participant reported being offered or receiving a referral, preconception consultation, or structured reproductive plan when personal barriers such as absence of a partner stood in the way of childbearing. One participant had received no fertility counseling at all.

**Discussion:**

These accounts left participants holding reproductive hopes they could not translate into plans. The findings suggest that at this facility fertility counseling is routinely delivered but, as experienced by these participants, incompletely comprehensive, and that structured individualized planning, provider capacity in safer conception, and clearly communicated referral pathways deserve attention.

## 1 Introduction

The global response to HIV has been transformed by the wide scale-up of antiretroviral therapy (ART), and an estimated 39.9 million people were living with HIV worldwide in 2023, with sub-Saharan Africa carrying the heaviest burden (1, 2). Effective treatment now allows people living with HIV/AIDS (PLWHA) who achieve and maintain viral suppression to reach a life expectancy that approaches that of the general population (3). This shift has reframed HIV from a fatal illness into a manageable chronic condition and has brought the reproductive aspirations of PLWHA to the center of comprehensive HIV care. Evidence from the PARTNER studies shows that among serodifferent couples in which the partner living with HIV was receiving effective ART and maintained a sustained undetectable viral load, no phylogenetically linked transmissions occurred across tens of thousands of condomless sex acts (4, 5). This finding, commonly summarized as undetectable equals untransmittable, applies specifically to individuals on effective treatment who achieve and sustain viral suppression, and it provides a conditional but powerful basis for supporting safe childbearing when that condition is met and monitored.

Against this background, a substantial proportion of PLWHA wish to have children. A systematic review and meta-analysis of 26 studies in Ethiopia estimated the pooled prevalence of fertility desire among PLWHA at 42.21% (95% confidence interval: 39.18–45.25), with a partner's desire for children strongly associated with fertility desire (6). Comparable patterns have been documented in Ghana, where women living with HIV report a continuing wish for children and frequently prefer to discuss reproductive concerns with their HIV clinicians (7). Ghana has made measurable progress in HIV testing coverage and treatment access, supported by national strategic frameworks, yet the country continues to record a considerable HIV burden and persistent service gaps (8–10). As survival and reproductive intentions rise together, the demand for accurate, supportive, and comprehensive fertility counseling within HIV care has intensified.

Comprehensive fertility counseling for PLWHA extends beyond the prevention of mother-to-child transmission (PMTCT). It encompasses safer conception strategies that protect HIV-negative partners, contraceptive options and their interactions with ART, individualized reproductive planning that reflects relationship circumstances and intentions, and timely referral to specialized services. Integrating reproductive health into HIV care is widely advocated because it positions providers to meet these needs at the point of routine contact (11). However, evidence accumulated over nearly two decades shows that such integration is often incomplete. Missed opportunities to address reproductive health needs during ART visits have been documented, and provider attitudes and limited safer conception knowledge frequently constrain the quality of counseling offered (12–14). Where intensified counseling and contraceptive services are delivered within HIV clinics, the benefits can be substantial, with one systematic review of 23 studies, mostly from sub-Saharan Africa, reporting that integrated provision increased subsequent modern contraceptive use by as much as fourfold (15). Despite this potential, unmet need for contraception and reproductive information persists among PLWHA across the region and in Ghana specifically (16, 17).

This study was framed by the Theory of Planned Behaviour, which holds that behavioural intentions are shaped by attitudes toward the behaviour, subjective norms arising from important others, and perceived behavioural control over whether the behaviour can be successfully performed (18). The theory was adopted as a sensitizing lens rather than as a coding framework. It informed the construction of the interview guide and the interpretation of findings by directing attention to how counseling might shape participants’ beliefs about childbearing, their sense of what significant others expect, and their confidence in being able to conceive and deliver safely. Its application to HIV care behaviours has been demonstrated in prior work (19). The conceptual framework guiding the study, presented in Figure 1, situates fertility intentions among PLWHA within socio-economic, cultural, and demographic influences, with the attitude of the health professional toward fertility intentions positioned as a cultural factor operating directly on the counseling encounter. In the Ghanaian context, patriarchal norms and partner pronatalism exert a powerful influence on women's fertility intentions, which makes that encounter a critical space in which information, reassurance, and planning can either enable or constrain safe reproduction (20).

**Figure 1:**
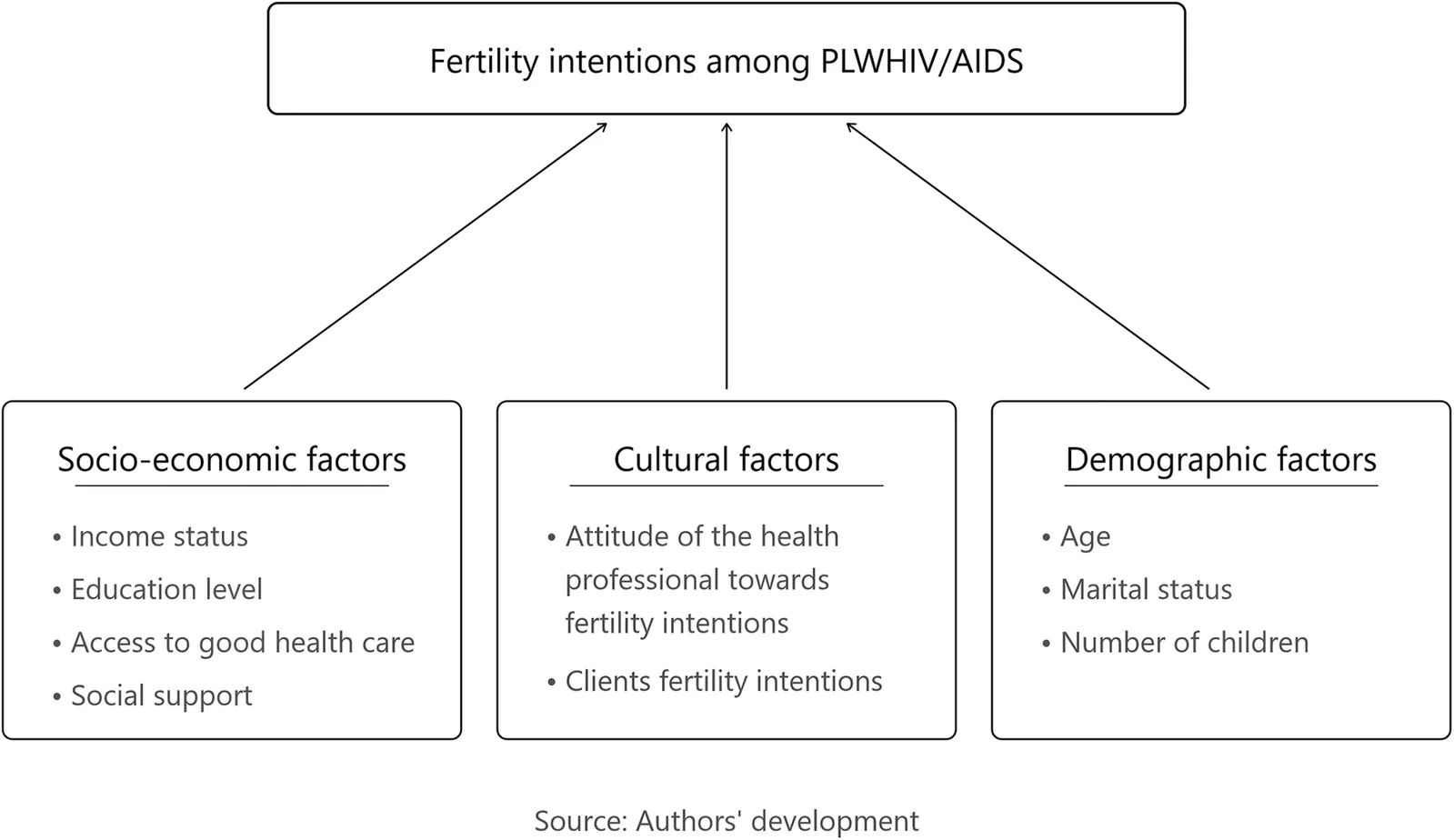
Conceptual framework of fertility intentions among PLWHIV/AIDS.

What remains unknown, and what this study addresses, is whether the fertility counseling actually delivered within Ghanaian ART care meets the standard of comprehensiveness described above, and where specifically it falls short from the perspective of those receiving it. Existing Ghanaian evidence is largely cross-sectional and contraception-focused, documenting how many women use a method and how many do not (7, 17), rather than describing what is said, and left unsaid, during counseling encounters. The content of counseling, the extent to which it is tailored to individual relationship and reproductive circumstances, whether it addresses protection of an HIV-negative partner during conception attempts, and what patients are told or offered when a barrier to childbearing is personal rather than clinical, have not been examined from the patient's own account in this setting. This study therefore explored the barriers to comprehensive fertility counseling among PLWHA attending an ART clinic in the Ashanti Region of Ghana, focusing on how the quality of counseling, provider attitudes, and access to reproductive health services shaped their fertility decision-making.

## 2 Materials and methods

### 2.1 Study design

This study adopted a qualitative design grounded in interpretive, or hermeneutic, phenomenology. Phenomenology seeks to understand individuals’ lived experiences and the meanings they attach to them, and it is well suited to research questions concerning deeply personal phenomena such as fertility decision-making with HIV (21). The interpretive tradition, in contrast to descriptive phenomenology, does not require the researcher to bracket prior understanding entirely. It treats the researcher's situated knowledge as a resource for interpretation that must be made explicit and held open to challenge, and it insists that experience be understood within its social, cultural, and institutional context (22, 23). This orientation was selected because what fertility counseling means to a person living with HIV cannot be separated from cultural expectations of parenthood, the relational context in which conception would occur, and the authority of the clinic in which counseling is delivered. Reporting followed the Consolidated Criteria for Reporting Qualitative Research, a 32-item checklist covering the research team, study design, and analysis and findings (24). The completed checklist is provided as Supplementary Material.

### 2.2 Study setting

The study was conducted at the ART clinic of St. Michael's Hospital, a Catholic district-level referral facility located at Pramso in the Bosomtwe District of the Ashanti Region, Ghana, approximately 20 kilometres from Kumasi along the Lake Bosomtwe road. The hospital provides comprehensive inpatient and outpatient services, including reproductive health care and a dedicated HIV/AIDS care clinic offering ART provision, viral load and CD4 monitoring, management of opportunistic infections, and counseling. At the time of the study, approximately 120 PLWHA were registered in the ART program and received free treatment in line with national guidelines. The facility was selected purposively because it serves a socioeconomically and culturally diverse catchment population of PLWHA in a region with a substantial HIV burden. Its status as a faith-based mission facility is relevant context, since institutional values may shape how reproductive topics are approached, and this is considered further in the Discussion. The study did not audit the facility's service inventory or referral protocols, so the reproductive services formally available at the site were not independently established, and findings on this point rest on what participants reported having been offered or received.

### 2.3 Participants and sampling

Participants were selected purposively. Eligibility was broad, comprising adults aged 18 years or older who were living with HIV, receiving ART at the clinic, and able to give informed consent. These criteria define eligibility rather than richness of experience, and the sample is therefore best described as a purposively selected group of clinic attendees rather than as an information-rich sample in the strict sense. Within this eligible pool, selection was guided by an intention to include variation in sex, age, marital status, and parity, on the reasoning that counseling needs and counseling experiences were likely to differ across these positions. The final sample comprised twelve participants, nine women and three men, aged 26–49 years.

Recruitment proceeded as follows. ART clinic nurses, who were not members of the research team, identified attendees meeting the eligibility criteria during routine visits and asked whether they were willing to hear about a study. Those who agreed were introduced to the first author, who then explained the study purpose, procedures, voluntary nature, and right to decline or withdraw, and obtained written informed consent in a private room. Clinic staff were not informed which of the individuals they introduced subsequently agreed to participate, were not present during any interview, and had no access to interview content. Participants were told explicitly and more than once, at introduction and again at consent, that participation, refusal, or withdrawal would have no bearing whatsoever on their treatment, medication supply, or standing at the clinic. The first author held no clinical or supervisory role at the facility and no responsibility for participants’ care, which was made clear to participants at the outset.

These measures were designed to reduce the risk that participants would feel obliged to take part or to speak favourably about the service. That risk cannot be eliminated in clinic-based recruitment, and the consequences for interpreting the findings are set out in Section 6, Limitations of the study.

### 2.4 Data collection

Data were collected over three months, from 4 August 2024 to 5 October 2024, through in-depth individual interviews. A semi-structured interview guide was developed from the research objectives and the literature on fertility among PLWHA, with question framing informed by the constructs of the Theory of Planned Behaviour. The guide covered participants’ diagnosis and HIV management experience, access to and experience of health care services, current thoughts and feelings about having children, reasons for wanting or not wanting children, the influence of HIV status on those thoughts, the factors that mattered most in reproductive decisions, how providers addressed their fertility concerns, whether they had received counseling specifically about fertility and HIV, and the barriers they faced. The guide was reviewed by the academic supervisor for clarity, coverage, and cultural appropriateness before fieldwork and was piloted with two clinic attendees who were not included in the analysed sample. Piloting led to minor changes in wording and sequencing, chiefly the placement of counseling questions after rapport-building questions about diagnosis and daily management. The guide was otherwise applied consistently, with probes used flexibly to follow participants’ accounts. The full guide is provided as Supplementary Material.

Interviews were conducted in English or Twi according to participant preference, in private rooms at the hospital, and lasted between 45 and 90 min. Interviews were audio-recorded with permission, and field notes documented contextual observations, non-verbal cues, and immediate analytic impressions.

Twi interviews were transcribed first in Twi, preserving the original wording, and were then translated into English by the first author, who is a native Twi speaker and a nurse-midwife familiar with the clinical and cultural idiom in which participants spoke. Transcription and translation were treated as sequential rather than simultaneous steps so that the original-language record remained available for return during analysis, an approach that reduces the loss of meaning associated with translating directly from audio (25). A second Twi-English bilingual colleague independently reviewed the English transcripts against the Twi originals, and discrepancies were resolved by discussion with reference to the recording. Where an expression carried a culturally specific meaning that had no direct English equivalent, the Twi term was retained in the transcript with an explanatory gloss rather than replaced by an approximate translation, since conceptual rather than literal equivalence is the appropriate standard in qualitative work (26). Analysis was conducted on the English transcripts with the Twi originals consulted whenever an interpretation turned on a specific phrase.

### 2.5 Data analysis

Analysis was conducted manually. No computer-assisted qualitative data analysis software was used. Transcripts were managed in word-processing and spreadsheet files, with coding recorded in margin annotations and consolidated in a coding matrix. Manual analysis was chosen deliberately, since the small dataset permitted close and repeated engagement with whole transcripts, which suits an interpretive phenomenological approach in which meaning is read within the context of an entire account rather than extracted as isolated segments.

Codes were generated inductively from participants’ own words. No pre-existing code list was imposed and the Theory of Planned Behaviour was not used to structure coding, though it informed later interpretation, so the analysis is best characterized as inductive with a theoretically informed interpretive phase. Initial line-by-line coding produced a set of descriptive codes that stayed close to participants’ language, using formulations such as fear of child infection, cost of child-rearing, and counseling not received. The full initial code list, together with the progression from initial codes through focused codes to sub-themes and themes, is provided as Supplementary Material.

The analytic process followed five interpretive phenomenological movements rather than a purely categorical thematic procedure. First, each transcript was read as a whole, repeatedly and alongside the audio, to grasp the participant's account in its own terms before any segmentation. Second, meaning units were delineated, being passages in which the participant expressed something significant about the experience of seeking or receiving fertility counseling. Third, each meaning unit was transformed from the participant's everyday expression into an interpretive formulation of what the experience appeared to mean for that person, so that a statement such as being taught at every visit was read not simply as a report of service frequency but as an experience of being addressed by the clinic in a particular way. Fourth, these interpretive formulations were synthesized within each transcript into a situated account of that individual's experience, preserving the coherence of the single case. Fifth, situated accounts were compared across participants to identify essential themes, meaning those structures of experience without which the phenomenon of incomplete fertility counseling would not be what it is. Movement between the parts and the whole was iterative throughout, in keeping with the hermeneutic circle.

Coding and interpretation were conducted primarily by the first author. The second author independently coded a subset of transcripts and reviewed the full coding matrix, the situated accounts, and the theme structure. Both authors met to compare interpretations, and disagreements, which concerned the boundary between two sub-themes within the provider attitudes theme, were resolved through return to the transcripts and reformulation of the sub-theme rather than by majority view. The academic supervisor reviewed the analytic framework at two points and challenged interpretations that appeared to run ahead of the data, which led directly to the reformulation described in Section 3.2.2.

### 2.6 Sample size and saturation

Sample size was not fixed in advance. Interviewing and preliminary analysis proceeded concurrently, and the decision to stop was based on the informational adequacy of the material for the research aim rather than on a target number. Saturation was tracked at two levels, following the distinction between code saturation, the point at which no new codes emerge, and meaning saturation, the point at which no new dimensions or nuances of an existing code emerge (27, 28).

Code saturation for the counseling-related material was reached by approximately the ninth interview, after which subsequent interviews generated no new descriptive codes relating to what counseling contained, how it was delivered, or what was missing from it. Meaning saturation required the full set, and the later interviews continued to add nuance, most importantly the account of a participant who had received no fertility counseling at all, which deepened rather than replicated the individualized planning code. Interviews eleven and twelve produced no new codes and no new dimensions within existing codes, and recruitment was stopped.

Saturation was assessed across the sample as a whole in relation to the counseling phenomenon, and this is a limitation rather than a strength. With nine women and three men, and with wide variation in age, marital status, and parity, the study was not powered to establish saturation within any sub-group. In particular, no claim of saturation is made for the accounts of men, for whom three interviews cannot support such a claim, nor for any particular relationship configuration. This constraint carries directly into how the findings are read. Wherever an interpretation in Section 3 or Section 4 rests wholly or partly on the accounts of male participants, it is presented as a reading of those individual accounts rather than as a characterization of men's counseling experience at this facility, and the text marks this explicitly at each point. What can be said overall is that the dimensions of the counseling experience described in this report recurred across the sample and stopped yielding new content, not that the experience of every sub-group has been exhaustively mapped. The constraint is revisited in Section 6, Limitations of the study.

### 2.7 Reflexivity and positionality

The first author is a female Ghanaian nurse-midwife and postgraduate researcher who trained and works in nursing and midwifery education in Kumasi. She is a Twi speaker from the study region and shares with most participants a cultural setting in which childbearing carries substantial social weight. She held no clinical or supervisory role at the study facility and was not involved in participants’ care. Her midwifery background gave her fluency in the clinical vocabulary of PMTCT and antenatal care, which eased rapport, and also carried the risk that she would hear participants’ accounts through a professional frame in which counseling is presumed to be beneficial and adequate. She entered the study with the prior expectation, formed in clinical training, that PLWHA would be reluctant to discuss childbearing. That expectation was not borne out and had to be revised, a revision recorded in the reflexive journal and one that materially changed how the individualized planning sub-theme was formulated.

The second author is a male biostatistician based at a teaching hospital and university outside the study region, with no prior relationship to the facility or its patients. His distance from the setting and his quantitative orientation were used deliberately as a counterweight during analysis, since he was positioned to question interpretations that relied on clinical assumption rather than on what participants had said. His disciplinary background carried its own risk, namely a tendency to treat recurrence across participants as the measure of significance, which sits uneasily with a phenomenological concern for the singular account. Both risks were named in analytic meetings.

Reflexivity was operationalized rather than merely asserted. A reflexive journal was kept from the first interview to the final theme structure, recording expectations before each interview, immediate impressions afterward, and the reasoning behind analytic decisions. Journal entries were revisited when interpretations were disputed. Attention was paid throughout to the possibility that participants were addressing the first author as a nurse and therefore moderating criticism of the clinic, and probes were framed to invite description of what had happened rather than evaluation of the service. Where an account was unusually positive, the field notes record whether the participant volunteered the assessment or produced it in response to a direct question, and this distinction informed how strongly such accounts were weighted.

### 2.8 Trustworthiness

Trustworthiness was pursued through strategies appropriate to interpretive qualitative work (29–31).

Credibility was supported by prolonged engagement across a three-month fieldwork period, by triangulation of transcripts with field notes, and by participant validation of preliminary interpretations. Member checking was conducted between the eighth week of fieldwork and the four weeks following its close. Eight of the twelve participants were available and agreed to take part. Two could not be reached and two declined on grounds of time. Participants were not returned full transcripts, which would have imposed a substantial reading burden and, in a study on a stigmatized condition, would have created a document in participants’ possession that carried disclosure risk. They were instead presented orally, in Twi or English as appropriate, with a summary of the situated interpretation of their own account and with the preliminary formulation of the themes, and were asked whether these recognized their experience (32). Seven participants confirmed the interpretations without amendment. One participant qualified the interpretation of her account, indicating that her reservations about childbearing had been shaped more by the absence of a partner than by concern about transmission, which had been the emphasis of the initial reading. This correction was accepted and is reflected in the formulation of the individualized planning sub-theme in Section 3.2.1. No participant proposed changes to the theme structure itself.

Dependability and confirmability were supported by an audit trail comprising the interview guide and its revisions, audio files, Twi and English transcripts, the initial code list, the coding matrix, situated accounts, reflexive journal entries, and dated records of analytic meetings and supervisory reviews. Transferability was supported by thick description of the setting, the participants, and the service context, so that readers may judge the applicability of the findings to their own settings.

### 2.9 Ethical considerations

Ethical approval was obtained from the Committee on Human Research, Publication and Ethics at the Kwame Nkrumah University of Science and Technology, College of Health Sciences, School of Dentistry (reference number CHRPE/AP/569/24), valid from 8 June 2024 to 7 July 2025. An introductory letter was received from the Kwame Nkrumah University of Science and Technology, College of Health Sciences (reference NURS/INTRO/Vol. 1, 7 March 2024). Additional ethical clearance was secured from the Catholic Health Service Trust-Ghana (reference number SMH/RA.007/24, received on 6 June 2024), and permission was obtained from the administration of St. Michael's Hospital.

All participants provided written informed consent after receiving detailed information about the study's purpose, procedures, risks, benefits, and their right to withdraw at any time without affecting their healthcare. Confidentiality was maintained through the use of participant codes (P1–P12) rather than names, secure storage of data on password-protected devices, and restriction of access to research team members only. Participants received no monetary compensation but were reimbursed for transportation costs incurred during study participation.

## 3 Results

### 3.1 Demographic characteristics of participants

The demographic characteristics of the twelve participants are presented in Table 1. The sample comprised nine women and three men whose ages ranged from 26 to 49 years, all of whom were living with HIV and receiving ART at the study clinic. Marital status included single, married, divorced, and widowed participants, allowing counseling experiences to be examined across a range of relationship contexts. Educational attainment varied from no formal education to tertiary level, employment situations ranged from student and unemployed to employed, and parity ranged from no children to six children.

**Table 1:** Demographic characteristics of participants.

Participant code	Age	Sex	Marital status	Education level	Employment status	Number of children	Children's HIV status
P1	26	Female	Single	Tertiary	Student	0	Not applicable
P2	38	Female	Divorced	Secondary	Unemployed	1	Negative
P3	27	Female	Married	Tertiary	Employed	1	Negative
P4	35	Male	Married	Secondary	Employed	2	All negative
P5	42	Female	Widow	None	Unemployed	2	All negative
P6	37	Female	Married	Tertiary	Employed	3	All negative
P7	49	Male	Married	Tertiary	Employed	6	All negative
P8	26	Female	Single	Tertiary	Student	0	Not applicable
P9	47	Female	Married	Secondary	Employed	3	All negative
P10	35	Female	Single	Tertiary	Employed	3	All negative
P11	28	Male	Single	Secondary	Employed	0	Not applicable
P12	33	Female	Married	Tertiary	Employed	2	All negative

As shown in Table 1, the age distribution spans the reproductive years, which placed the sample well to reflect on counseling needs at different life stages, and the inclusion of both nulliparous and multiparous participants allowed the influence of parity on counseling experiences to be explored. Table 1 also records that the children of all participants who had children were HIV-negative. This observation is reported here as an attribute of the sample and no inference about the effectiveness or coverage of PMTCT services is drawn from it, for reasons set out below. Age at diagnosis and duration of ART were not systematically recorded at data collection and are therefore not reported in Table 1. Their absence is acknowledged as a limitation in Section 6, Limitations of the study, since time since diagnosis plausibly shapes both the counseling a person has accumulated and the reproductive stage at which they encountered it.

The reason for interpretive caution about the HIV-negative status of participants’ children is set out in Table 2. Whether a child's negative status carries any information about PMTCT depends entirely on whether that child was conceived and born after the mother's diagnosis and ART initiation. Where a child was born before the mother knew her status, the child's negative result reflects the absence of maternal infection at the time rather than the success of any intervention.

**Table 2:** Reported timing of HIV diagnosis relative to childbirth among participants with children.

Participant	Number of children	Reported timing of diagnosis relative to birth	Basis for classification
P2	1	Child born before diagnosis	Participant stated the child was born prior to acquiring the infection
P3	1	Not documented	Timing not addressed in the interview
P4	2	Not documented	Timing not addressed in the interview
P5	2	Not documented	Timing not addressed in the interview
P6	3	Diagnosed during a pregnancy	Participant was diagnosed at a routine antenatal visit
P7	6	Children preceded diagnosis	Participant described being untroubled by diagnosis because his family was already complete
P9	3	Children preceded diagnosis	Participant described being unaffected because she already had all her children
P10	3	At least one child born after diagnosis	Participant was diagnosed while pregnant and reported the baby was not affected
P12	2	Not documented	Timing not addressed in the interview

Table 2 makes clear why the earlier inference could not be sustained. For P7 and P9, whose children preceded diagnosis, the negative status of those children says nothing about PMTCT. For P2, the single child was likewise born before infection. Only P10, diagnosed during pregnancy and reporting that the baby was subsequently unaffected, offers an account in which PMTCT interventions plausibly operated, and P6 was similarly diagnosed at an antenatal visit. For four participants the timing was not documented. On this evidence the sample cannot speak to PMTCT effectiveness or coverage, and Table 2 is presented not as a finding about PMTCT but as a boundary marker on what the demographic data in Table 1 can and cannot support.

### 3.2 Thematic structure of healthcare experiences shaping fertility counseling

Interpretation of participants’ situated accounts produced three essential themes and nine sub-themes, summarized in Table 3. The three themes were quality of reproductive health counseling and education, healthcare provider attitudes and support, and access to comprehensive reproductive health services. These themes capture both the elements of care that participants experienced as sustaining and the gaps that left their reproductive hopes unsupported.

**Table 3:** Themes and sub-themes on healthcare experiences shaping fertility counseling.

Main Theme	Sub-themes
Theme 1: Quality of Reproductive Health Counseling and Education	1.1 Comprehensive PMTCT education; 1.2 Safer conception counseling for serodifferent couples; 1.3 Individualized reproductive health planning
Theme 2: Healthcare Provider Attitudes and Support	2.1 Supportive and encouraging provider approaches; 2.2 Counseling misaligned with settled reproductive intentions; 2.3 Provider comfort with fertility discussions
Theme 3: Access to Comprehensive Reproductive Health Services	3.1 ART access and adherence support; 3.2 Integration of reproductive health into HIV care; 3.3 Reported access to specialized reproductive services

Table 3 shows that the counseling encounter was constituted for participants along three dimensions. The first concerns what was said, the second concerns the manner and relational climate in which it was said, and the third concerns what participants understood the service to offer once something had been said. Read together, the sub-themes in Table 3 indicate that the incompleteness participants experienced did not arise from a single deficiency. Strong and confidently delivered PMTCT messaging coexisted with counseling that was not adjusted to the individual, with variation in how proactively fertility was raised, and with an absence of any onward option that participants were aware of or had been offered.

Quotations in Tables 4–6 are drawn from the accounts of eight participants whose narratives addressed fertility counseling directly and in sufficient detail. All twelve participants contributed to code development and to the construction of the theme structure. The remaining four participants discussed diagnosis, treatment, and childbearing intentions but did not describe their counseling encounters in terms that could be quoted meaningfully against these sub-themes, and no attempt was made to press their accounts into illustrative service. Of the three men in the sample, one is quoted, and readings that rest on his account are identified as such in the text and are not generalized to men attending the clinic.

**Table 4:** Illustrative accounts for quality of reproductive health counseling and education.

Sub-theme	Illustrative accounts	Participant
1.1 Comprehensive PMTCT education	“That having the disease doesn't deter one from not having kids and in our condition we can still have healthy babies.”	P8, Female, Single
	“That even if I want to have a child again, taking my medications will not affect the baby.”	P9, Female, Married
1.2 Safer conception counseling for serodifferent couples	“There are drugs I can take to prevent my baby from having the disease and how I can get pregnant with my partner without infecting him as well.”	P2, Female, Divorced
	“That I can still give birth in my condition without affecting my child.”	P6, Female, Married
1.3 Individualized reproductive health planning	“Not yet, but I think along the line I will receive counselling on fertility issues.”	P1, Female, Single
	“We are being taught every time I visit the hospital and I will rate them about 70%. I want to have more babies but I don't have a partner at the moment.”	P2, Female, Divorced

**Table 5:** Illustrative accounts for healthcare provider attitudes and support.

Sub-theme	Illustrative accounts	Participant
2.1 Supportive and encouraging provider approaches	“I have a very cordial experience with the health workers, I am saying so because they granted me unlimited access to have time with them and also have time for me.”	P1, Female, Single
	“They are friendly, confidential and patient with me.”	P6, Female, Married
2.2 Counseling misaligned with settled reproductive intentions	“Yes but I don't pay much attention because I have enough kids.”	P7, Male, Married
	“Yes we are taught but I don't need babies again so I don't really pay attention to it.”	P9, Female, Married
2.3 Provider comfort with fertility discussions	“Yes please, every time I come for checkup.”	P8, Female, Single
	“Yes they do and we are been encouraged to have children and live our lives as any other person.”	P3, Female, Married

**Table 6:** Illustrative accounts for access to comprehensive reproductive health services.

Sub-theme	Illustrative accounts	Participant
3.1 ART access and adherence support	“I have access and by taking the medications given I feel healthy.”	P8, Female, Single
	“I receive all the necessary services and I have easy access to all my health care needs.”	P3, Female, Married
3.2 Integration of reproductive health into HIV care	“We are being taught every time I visit the hospital … I am counselled each time I visit the hospital.”	P2, Female, Divorced
	“Yes please, we are taught at the hospital after child bearing in our condition and our general wellbeing.”	P3, Female, Married
3.3 Reported access to specialized reproductive services	“I want to have more babies but I don't have a partner at the moment. I am hesitant in child bearing but hopefully one day I will follow all the processes so I can get a healthy baby.”	P2, Female, Divorced
	“I want a baby but doesn't want to put him/her at risk because of the disease.”	P6, Female, Married

#### 3.2.1 Quality of reproductive health counseling and education

Illustrative accounts for the first theme are presented in Table 4.

The accounts in Table 4 reveal a counseling experience that was confident at its centre and thin at its edges. For P8, whose account appears under sub-theme 1.1, what the clinic told her did not simply add information. It removed a prohibition. Her formulation that the disease does not deter one from having children carries the structure of a permission granted, and she went on to describe hoping that when the time came she would carry to term and deliver a healthy baby as any other woman would. The phrase as any other woman discloses what was at stake for her, which was not obstetric risk but membership in an ordinary category of womanhood from which she had feared exclusion. Counseling here worked at the level of identity, restoring a future she had assumed was closed. P9's statement in Table 4 does comparable work in a different register, converting her medication from a threat to the baby into something compatible with pregnancy, so that treatment ceased to stand between her and any further childbearing.

Sub-theme 1.2 in Table 4 shows where that confidence stopped. P2 had received the fuller package, and her account names both objects of protection, the baby and the partner. What this meant for her was that conception could be imagined as a manageable procedure rather than a gamble, and she spoke of following all the processes so as to get a healthy baby, a phrase in which childbearing has become a sequence with steps she could in principle follow. P6's account in Table 4 shows the more common shape. Her counseling addressed giving birth in her condition without affecting her child, and it stopped there. The partner does not appear. P6 was married and described being under pressure from her husband to have another child, and elsewhere she said she wanted a baby but did not want to put the child at risk. The counseling she described spoke to the second of those concerns and was silent on the relational situation in which conception would actually take place. The consequence was not that she rejected childbearing but that she remained suspended, wanting and hesitating at once, without information that addressed the encounter with her husband through which any pregnancy would come about.

Sub-theme 1.3 in Table 4 contains the study's clearest evidence of counseling that did not reach the participant. P1 had received no fertility counseling. Her formulation is notable for its patience rather than its complaint, since she expected that counseling would come along the line, and she described providers as giving her drugs to that effect when asked how they addressed her fertility concerns. She was a childless single woman of 26 who said she wished for children while fearing they would be infected, and who had concluded that she intended not to have children. That conclusion had been reached without any counseling encounter in which the fear driving it could be examined. What her account discloses is the cost of counseling that is delivered but not directed, since the information given to P8 and P9 would have spoken directly to P1's fear had it reached her. P2's account in the same sub-theme shows the parallel gap for someone who was being counselled regularly. She was taught at every visit, rated the service at about seventy per cent, and identified her actual obstacle as having no partner following her husband's departure. Regular counseling had not engaged the one circumstance standing between her intention and its realization, and her rating below excellence should be read as a precise verdict on that mismatch rather than as general dissatisfaction.

#### 3.2.2 Healthcare provider attitudes and support

Accounts relating to provider attitudes and support are summarized in Table 5. This sub-theme structure was reformulated during analysis. An earlier formulation grouped accounts under perceived judgmental attitudes, but return to the transcripts established that no participant described a provider behaving, speaking, or appearing judgmental toward their fertility intentions. What the accounts described was participants’ own disengagement from counseling they regarded as irrelevant to settled intentions, which is a different phenomenon, and the sub-theme was renamed accordingly.

The accounts in Table 5 describe a relational climate that participants experienced as genuinely hospitable. P1's account under sub-theme 2.1 is striking precisely because it comes from the participant who had received no fertility counseling. She experienced providers as granting unlimited access and as having time for her, and her emphasis on their having time for me suggests that what she valued was being treated as a person deserving unhurried attention rather than as a case to be processed. The coexistence of that warmth with the complete absence of fertility counseling is the significant finding here. Being well received and being reproductively counselled were, for her, separate things, and the first did not deliver the second. P6's account of providers as friendly, confidential, and patient names confidentiality specifically, which for a woman whose husband had left her after her diagnosis indicates that the clinic functioned as a space where her status did not travel.

Sub-theme 2.2 in Table 5 requires careful reading, and a caution about scope applies before the reading begins. P7 is one of only three men in the sample and the only man quoted, and as set out in Section 2.6 no claim of saturation is made for male participants. What follows is therefore an interpretation of two individual accounts, one from a man and one from a woman, and it is not offered as a characterization of how men at this clinic experience fertility counseling. P7, a man with six children, said he did not pay much attention because he had enough children, and P9 said she was taught but did not need babies again and so did not really attend to the teaching. Neither statement describes anything a provider did. Both describe the participant's own withdrawal of attention from material that did not correspond to their reproductive position. For P7 this sat within a broader account in which HIV had not disturbed his life, since he had not been worried at diagnosis for the same reason, namely that his family was complete. What these two accounts disclose about the counseling they received is that it was delivered without regard to their stated intention, and that both, having settled their reproductive positions, experienced it as something to be sat through. This is a reading about the calibration of counseling as these two participants encountered it rather than about provider attitude. It is possible that a provider assumption about parity underlies the pattern, but no participant said so, and that possibility is not asserted here. Whether the pattern extends to other men at this facility cannot be determined from three male interviews.

Sub-theme 2.3 in Table 5 indicates that providers did raise fertility without waiting to be asked. P8 received counseling at every checkup, and P3 described being encouraged to have children and to live as any other person would. The phrase live our lives as any other person recurs in form with P8's as any other woman, and the repetition across two participants suggests that a normalizing message was being communicated deliberately at this clinic. What this meant for those who received it was a lifting of the assumption that HIV had disqualified them from ordinary reproductive life. P3 said the counseling had changed her perspective on marriage and childbearing, which is the strongest statement of transformation in the dataset. Set against P1's silence, the picture is of a clinic capable of delivering a message that changes how people see their futures, and not yet reaching everyone with it.

#### 3.2.3 Access to comprehensive reproductive health services

Illustrative accounts for the third theme are presented in Table 6.

The accounts in Table 6 show that the foundation of the service was secure and that what sat above it was, for these participants, undefined. Under sub-theme 3.1, P8's linking of medication access to feeling healthy is the precondition on which every reproductive hope in this dataset rests, since a body experienced as failing does not entertain pregnancy. What ART gave her was not merely clinical stability but the felt sense of being well, and it was from within that sense that childbearing became thinkable. P3's account of easy access to all her health care needs speaks to the same reliability of provision.

Sub-theme 3.2 in Table 6 confirms that reproductive topics were built into the routine visit rather than left to patient initiative. P2 was counselled each time she attended, and P3 located reproductive teaching within general wellbeing education, indicating that fertility was not sequestered as a specialist topic. This structural integration is a real strength of the service and is the platform on which the missing elements could be added.

Sub-theme 3.3 in Table 6 concerns what integration alone did not resolve for two participants. The scope of this sub-theme requires precision. Both illustrative accounts come from women, P2 and P6, describing their own fertility circumstances, and what they report is that no referral, specialist consultation, or structured reproductive plan was offered to or discussed with them. The study did not audit the facility's service inventory or referral protocols, and no inference is drawn here that specialized reproductive services were unavailable at St. Michael's Hospital or in the wider district. The finding is about what these participants were told and offered, not about what the facility holds. P2 wanted more children, had no partner, was hesitant, and yet held to the hope of one day following all the processes. The temporal structure of her account is the finding, since her plan had no identified next step and existed only as a deferred someday, and nothing she described in the service had addressed the transition from her present situation to the processes she believed would eventually be available to her. P6's account has the same suspended quality from the opposite direction, since she had a partner and wanted a child but could not resolve the risk to that child, and neither woman described being offered a referral, a fertility assessment, or a planned preconception consultation. What this meant experientially was therefore not the denial of a procedure. It was that neither woman could identify anyone to take the next step with, so that their reproductive intentions remained private hopes rather than becoming clinical plans. Whether this reflects an absence of services, an absence of referral practice, or an absence of communication about services that exist cannot be determined from patient accounts alone.

## 4 Discussion

This study explored the barriers to comprehensive fertility counseling among PLWHA receiving ART at a district ART clinic in the Ashanti Region of Ghana. Participants described counseling that was routinely delivered, relationally warm, and structurally integrated into HIV care, yet incompletely comprehensive in three specific respects. It was calibrated to the population rather than to the individual, it addressed the child but seldom the partner, and no participant reported being offered an onward pathway when the obstacle to childbearing lay outside the clinical domain. One participant had received no fertility counseling at all despite regular attendance and a warm relationship with providers.

### 4.1 PMTCT education as an enabler and the limits of undirected provision

Participants who received PMTCT education described a restoration of reproductive possibility, which aligns with evidence that provider education is a crucial enabler of reproductive realization for PLWHA (12, 13). Interpreted through the sensitizing lens of the Theory of Planned Behaviour, this material speaks to perceived behavioural control, since concrete information about risk reduction allowed participants to envisage safe pathways to childbearing that had previously seemed closed (18). It also speaks to attitude, since the normalizing message that PLWHA may live and bear children as any other person does works on the evaluation of childbearing itself rather than only on its perceived feasibility.

The accounts of P7 and P9, however, should not be read as evidence of a knowledge to confidence gap. Their withdrawal of attention was not a failure of reassurance but an accurate judgement that the material did not concern them, since both had completed their families and neither wanted further children. Counseling that is delivered uniformly to a population with heterogeneous reproductive intentions will necessarily be irrelevant to a portion of its audience, and that irrelevance is a design characteristic rather than a psychological deficit in the recipient. Because P7 is one of only three men interviewed and no claim of saturation is made for male participants, this reading rests on two individual accounts and is not advanced as a gendered pattern. The substantive problem it reveals is the reciprocal one seen in P1, namely that a service which does not assess intention cannot direct its content, and so simultaneously over-serves those who have finished childbearing and under-serves a childless young woman who had resolved against children on the basis of a fear no one had addressed. Given a pooled fertility desire prevalence of 42.21% among PLWHA in the Ethiopian meta-analysis, with wide variation across settings, the case for assessing intention before counseling rather than counseling in its absence is a strong one (6). Family planning counseling delivered within HIV services has been shown to be effective when it is intensified and supported, which suggests the difficulty here is one of targeting rather than of the intervention itself (15).

### 4.2 Counseling that addressed the child but not the partner

The most consistent content gap was the near absence of safer conception counseling addressing protection of an HIV-negative or unknown-status partner. Only P2 described having received it. P6, married and under pressure from her husband to conceive, had been counselled about protecting a future child and not about the conception itself. This gap matters because the PARTNER studies established that among serodifferent couples in which the partner living with HIV is on effective ART and maintains sustained viral suppression, no linked transmissions were observed across many thousands of condomless acts (4, 5). Properly qualified, this evidence is precisely what a woman in P6's position needed, since it converts the question from whether conception is dangerous into whether her viral suppression is sustained and monitored. Its absence from her counseling left her without the information that would have resolved her suspension.

This under-provision mirrors findings across sub-Saharan Africa, where provider knowledge of safer conception has been limited and such discussions rare, constraining the reproductive choices of HIV-affected couples (14). Closing the gap carries particular weight in Ghana given that a partner's desire for children is among the strongest correlates of fertility desire among PLWHA, with the Ethiopian meta-analysis reporting an odds ratio of 16.8 (6), and given the documented influence of partner pronatalism on women's fertility intentions in this country (20). In the Kumasi study of women living with HIV, contraceptive use was 93% among women with HIV-positive partners but 65% and 67% among those with HIV-negative and untested partners respectively, and partner preference was the single most common reason for non-use, cited by 46% of non-users, with an overall unmet need for contraception of 10% and 26% of sexually active women using no method (7). Those figures describe a reproductive landscape organized around the partner, which makes counseling that omits the partner structurally incomplete. Comparable patterns of unmet reproductive need among PLWHA have been reported in Tanzania and Kenya and in Ghanaian contraceptive research (16, 17, 33, 34), and fertility desire among ART users remains substantial across East African programs (35). The present study is not positioned to comment on how men living with HIV in this setting encounter partner protection counseling, since only three men were interviewed and only one addressed counseling content in a quotable form.

### 4.3 Relational warmth as a necessary but insufficient condition

Supportive provider relationships emerged clearly, consistent with evidence that non-judgmental attitudes encourage engagement while perceived judgment creates barriers to disclosure and service use (13). Notably, this study found no participant account of provider judgment, which differs from settings where discouraging attitudes toward childbearing by PLWHA have been documented (13, 14), and which may reflect genuine local practice or may reflect participants’ reluctance to criticize a service on which they depend.

The more consequential finding is the dissociation between warmth and counseling content revealed by P1, who experienced providers as generous with time and had received no fertility counseling whatsoever. A hospitable clinical relationship does not by itself generate reproductive counseling, and the two must be separately assured. Cooper and colleagues argued for integrating reproductive health into HIV care on the reasoning that integration positions providers to meet these needs at the point of routine contact (11), and this study suggests that positioning is necessary but not sufficient, since contact and even rapport occurred without the counseling following.

The faith-based character of the study facility warrants mention as context. Mission facilities operate within institutional value frameworks that may shape how conception outside marriage, contraception, or assisted reproduction are approached. No participant attributed any omission to institutional doctrine and no such attribution is made here, but the setting is relevant to how these findings should be read and to their transferability.

### 4.4 Integration without a communicated onward pathway

Integration of reproductive counseling into routine visits was a real structural strength, and integrated provision has been shown to increase modern contraceptive use by as much as fourfold across 23 studies, mostly from sub-Saharan Africa (15). Reliable ART access underpinned participants’ sense of bodily wellness, reflecting the way effective treatment, which now supports a life expectancy approaching that of the general population, reshapes what people permit themselves to want (3).

What integration did not supply, for the two women whose accounts anchor this finding, was a next step they could identify. Both P2 and P6 described intentions suspended in an indefinite future with no articulated action between their present situation and its realization, and neither described being offered a referral, a fertility assessment, a planned preconception consultation, or a scheduled review of reproductive intentions. This is a statement about what these participants reported being offered, and it should not be read as establishing that such services were unavailable at the facility. Patient accounts can show that a pathway was not communicated or not taken up; they cannot show that no pathway exists. The distinction matters for how the finding should be acted upon, since an absent service and an available service that patients are never told about call for different remedies, and the present data cannot separate the two. What the accounts do establish is that the experiential meaning of this gap was the absence of a partner in planning rather than the denial of a technology, which suggests that the first remedy to test is a structured reproductive planning conversation with defined follow-up rather than the provision of assisted reproductive technology.

## 5 Implications

The implications set out here are derived from the accounts of twelve participants at a single district-level, faith-based ART clinic and should be read as propositions arising from that setting rather than as conclusions about PLWHA in Ghana generally. Their applicability elsewhere is a matter for readers to judge against their own contexts, and testing them more widely is a task for future research. Because only three men were interviewed and saturation was not established within that sub-group, none of the propositions below is offered as addressing men's counseling needs specifically.

Within the study setting, the findings suggest four directions. First, a brief structured assessment of reproductive intention at ART review would allow counseling to be directed rather than broadcast, which would address both the irrelevance experienced by participants with completed families and the omission experienced by a childless participant who had received nothing. Second, counseling content would benefit from routinely extending to the conception event itself, including a properly qualified account of the relationship between sustained viral suppression on effective ART and transmission risk to a partner (4, 5). Third, provider capacity in safer conception, both knowledge and communicative comfort, appears to warrant strengthening, consistent with regional evidence on provider knowledge limitations (14). Fourth, since no participant reported being offered or informed of an onward pathway, a useful first step would be to establish what reproductive referral options the facility and district already hold and then ensure they are actively communicated and documented at the point of counseling. Whether the gap these participants experienced calls for creating a pathway or for communicating an existing one is a question a service audit would settle, and it should precede any decision to develop new provision. These elements could be embedded within the integrated platform participants already valued, which offers a feasible route rather than requiring a new service (11, 15).

## 6 Limitations of the study

Several limitations qualify these findings.

The study was conducted at a single district-level, faith-based ART clinic with twelve participants. The findings are context-specific and are not offered as representative of PLWHA in Ghana. Thick description of the setting is provided so that readers may assess transferability to their own contexts.

The sample was skewed by sex, comprising nine women and three men. This is a material limitation given how substantially the analysis engages partner dynamics and differences in counseling content. Men's experiences of fertility counseling, their role in couple-level reproductive decisions, and their exposure to safer conception information cannot be adequately characterized from three accounts, and the male perspective on partner protection counseling in particular remains under-described. Findings relating to partner dynamics are therefore weighted toward women's accounts of their partners rather than toward men's accounts of themselves. Only one man is quoted in the results, and the readings that draw on his account are marked in Sections 3.2.2 and 4.1 as interpretations of an individual account rather than as findings about men at this facility.

Saturation was assessed for the counseling phenomenon across the sample as a whole and not within sub-groups. No claim of saturation is made for men, for any particular relationship configuration, or for any parity group.

The study did not audit the facility's reproductive service inventory, referral protocols, or counseling records. Findings concerning specialized services and referral therefore describe what participants reported having been offered or received, and cannot establish what the facility provides. An apparent gap in this study may reflect an absent service, an absent referral practice, or an available service that was not communicated to these participants, and the design cannot distinguish between these.

Age at diagnosis and duration of ART were not systematically collected. These variables plausibly shape both the volume of counseling a participant has accumulated and the reproductive stage at which it was encountered, and their absence limits interpretation of variation between participants.

Recruitment took place within the clinic where participants received ongoing care. Although clinic staff were not research team members, did not know who consented, and were absent from interviews, and although participants were repeatedly assured that participation would not affect their care, selection and social desirability bias cannot be excluded. Participants who were content with the service may have been more willing to take part, and participants may have moderated criticism when speaking with a researcher who is a nurse-midwife. The generally positive accounts of provider support should be read with this in mind, as should the absence of any account of provider judgment.

The study captured patients’ perspectives only. Provider and system-level drivers of the barriers identified, including training, caseload, time per consultation, and institutional policy, were not examined.

Quotations are drawn from eight of the twelve participants, since four did not describe their counseling encounters in terms that could be quoted meaningfully against the sub-themes, though all twelve informed code and theme development.

Finally, most interviews were conducted in Twi and analysed in English translation. Although sequential transcription and translation, independent bilingual review, and retention of untranslatable terms were used to preserve meaning, some nuance is lost whenever interpretation crosses languages (25, 26).

## 7 Conclusion

At the ART clinic studied, fertility counseling was routinely delivered, warmly received, and integrated into ordinary HIV care, and it was nonetheless incomplete in ways that mattered to the people receiving it. Participants were told that HIV need not end their reproductive lives, and several described that message as transforming how they saw their futures. What they were not given was counseling calibrated to their own intentions, information about protecting a partner during conception, or any next step they could name when the obstacle they faced was relational rather than clinical. One participant, well regarded by her providers and attending regularly, had received no fertility counseling at all and had decided against children on the basis of an unexamined fear. These findings suggest that within this setting the task is less to introduce fertility counseling than to complete it, through assessment of reproductive intention, content that reaches the conception event and the partner, provider capacity in safer conception, and referral options that are established, communicated, and documented so that intentions can become plans. Because the study drew on patient accounts alone, and on only three male accounts, whether the same pattern holds for men at this facility and in other Ghanaian settings remains open, and multi-site, provider-inclusive research that also audits available services would be well placed to answer it.

## Data Availability

The qualitative datasets generated and analysed during this study contain sensitive and potentially identifying information and are therefore not publicly available, in order to protect participant confidentiality. De-identified excerpts and the coding matrix may be made available by the corresponding author on reasonable request and subject to ethical approval.
