# Supplementary material for: Barriers to comprehensive fertility counseling among persons living with HIV/AIDS in Ghana
Source: Front Reprod Health. 2026 Oct 16;8:1914739. doi: 10.3389/frph.2026.1914739 (PMC13719461; doi:10.3389/frph.2026.1914739)
Supplement: Supplementary file 1 [file Datasheet1.docx]

**SUPPLEMENTARY DATA SHEET 1**

**Consolidated Criteria for Reporting Qualitative Research (COREQ): 32-item checklist**

**Manuscript:** Barriers to Comprehensive Fertility Counseling Among Persons Living with HIV/AIDS in Ghana

**Authors:** Priscilla Asantewaa Boadi, Victor Luckyboy Dzramado

**Journal:** Frontiers in Reproductive Health, HIV and STIs specialty section. Article type: Original Research.

**Checklist source:** Tong A, Sainsbury P, Craig J. Consolidated criteria for reporting qualitative research (COREQ): a 32-item checklist for interviews and focus groups. Int J Qual Health Care. 2007;19(6):349-357.

***Note on use.*** *Section numbers in the final column refer to the numbered sections of the revised manuscript. Where an item was not addressed in the study, this is stated plainly rather than left blank, since COREQ is a reporting standard and an item that was not done is reported as such.*

| **No.** | **Topic and guide question** | **How the item is addressed in this manuscript** | **Location** |
| --- | --- | --- | --- |
| **Domain 1: Research team and reflexivity** | | | |
| ***Personal characteristics*** | | | |
| 1 | **Interviewer / facilitator**  *Which author(s) conducted the interview or focus group?* | All twelve in-depth individual interviews were conducted by the first author, Priscilla Asantewaa Boadi (PAB). The second author (VLD) conducted no interviews and had no contact with participants at any stage. | Sections 2.4, 2.7 |
| 2 | **Credentials**  *What were the researcher's credentials?* | PAB is a registered nurse-midwife and postgraduate researcher (MPhil candidate) in nursing and midwifery.  VLD is a biostatistician holding a first degree in statistics and a postgraduate qualification in epidemiology and biostatistics.  The study was supervised by an academic supervisor who reviewed the analytic framework at two points. | Section 2.7 |
| 3 | **Occupation**  *What was their occupation at the time of the study?* | PAB was employed in nursing and midwifery education in Kumasi and held no clinical or supervisory role at the study facility.  VLD was employed as a biostatistician at a university department and teaching hospital outside the study region. | Sections 2.3, 2.7 |
| 4 | **Gender**  *Was the researcher male or female?* | PAB (interviewer and lead analyst) is female.  VLD (second coder and corresponding author) is male.  Both are identified by gender in the reflexivity and positionality section. | Section 2.7 |
| 5 | **Experience and training**  *What experience or training did the researcher have?* | PAB holds midwifery training and clinical familiarity with the vocabulary of prevention of mother-to-child transmission and antenatal care, together with postgraduate research training in qualitative methods. She is a native Twi speaker from the study region.  VLD contributed methodological and analytic expertise in health research and independently coded a subset of transcripts.  The interview guide was reviewed by the academic supervisor and piloted with two clinic attendees before fieldwork. | Sections 2.4, 2.5, 2.7 |
| ***Relationship with participants*** | | | |
| 6 | **Relationship established**  *Was a relationship established prior to study commencement?* | No prior relationship existed. PAB held no clinical or supervisory role at St. Michael's Hospital and carried no responsibility for participants' care. Participants were introduced to her by ART clinic nurses who were not members of the research team, and first met her at the point of study explanation and consent. | Sections 2.3, 2.7 |
| 7 | **Participant knowledge of the interviewer**  *What did the participants know about the researcher?* | At introduction and again at consent, participants were informed of the study's purpose, procedures, voluntary nature, and their right to decline or withdraw. They were told explicitly and more than once that participation, refusal, or withdrawal would have no bearing on their treatment, medication supply, or standing at the clinic, and that the researcher held no clinical role at the facility and no responsibility for their care. | Sections 2.3, 2.9 |
| 8 | **Interviewer characteristics**  *What characteristics were reported about the interviewer? e.g. bias, assumptions, reasons and interests in the research topic* | Reported in a dedicated positionality section. PAB's midwifery background eased rapport but carried the risk of hearing accounts through a professional frame in which counseling is presumed adequate. She entered fieldwork expecting that persons living with HIV would be reluctant to discuss childbearing; this expectation was not borne out, and its revision is documented in the reflexive journal and materially changed the formulation of sub-theme 1.3.  VLD's quantitative orientation carried a countervailing risk of treating recurrence across participants as the measure of significance. Both risks were named in analytic meetings and used as mutual checks.  Probes were framed to invite description of events rather than evaluation of the service, and field notes recorded whether positive assessments were volunteered or elicited. | Sections 2.5, 2.7, 2.8 |
| **Domain 2: Study design** | | | |
| ***Theoretical framework*** | | | |
| 9 | **Methodological orientation and theory**  *What methodological orientation was stated to underpin the study?* | The study is grounded in interpretive (hermeneutic) phenomenology. The analytic process is described as five interpretive phenomenological movements, namely holistic reading, delineation of meaning units, transformation into interpretive formulations, synthesis into situated accounts, and cross-case identification of essential themes, with iterative movement between parts and whole in keeping with the hermeneutic circle.  The Theory of Planned Behaviour is introduced in the Introduction as a sensitizing lens informing the interview guide and the interpretive phase. It was expressly not used as a coding framework, and coding was inductive. | Introduction; Sections 2.1, 2.5 |
| ***Participant selection*** | | | |
| 10 | **Sampling**  *How were participants selected?* | Purposive selection. Eligibility comprised adults aged 18 years or older, living with HIV, receiving ART at the clinic, and able to give informed consent. Within this eligible pool, selection was guided by an intention to include variation in sex, age, marital status, and parity.  The manuscript states explicitly that these criteria define eligibility rather than richness of experience, and describes the sample as a purposively selected group of clinic attendees rather than an information-rich sample in the strict sense. | Section 2.3 |
| 11 | **Method of approach**  *How were participants approached?* | Face to face, in two stages. ART clinic nurses who were not members of the research team identified eligible attendees during routine visits and asked whether they were willing to hear about a study. Those who agreed were introduced to PAB, who explained the study and obtained written informed consent in a private room. Clinic staff were not told which of the individuals they introduced subsequently consented. | Section 2.3 |
| 12 | **Sample size**  *How many participants were in the study?* | Twelve participants, comprising nine women and three men, aged 26 to 49 years. Sample size was not fixed in advance; interviewing and preliminary analysis proceeded concurrently and recruitment stopped on informational grounds. | Sections 2.3, 2.6, 3.1; Table 1 |
| 13 | **Non-participation**  *How many people refused to participate or dropped out? Reasons?* | The number of eligible attendees who declined at the introduction stage was not recorded during fieldwork and is therefore not reported. No participant withdrew after providing consent, and all twelve interviews were completed and analysed.  At the member-checking stage, eight of the twelve participants took part; two could not be reached and two declined on grounds of time. | Sections 2.3, 2.8 |
| ***Setting*** | | | |
| 14 | **Setting of data collection**  *Where was the data collected?* | In private rooms at the ART clinic of St. Michael's Hospital, a Catholic district-level referral facility at Pramso in the Bosomtwe District of the Ashanti Region, Ghana, approximately 20 kilometres from Kumasi. At the time of the study approximately 120 persons living with HIV were registered in the ART programme and received free treatment in line with national guidelines. | Sections 2.2, 2.4 |
| 15 | **Presence of non-participants**  *Was anyone else present besides the participants and researchers?* | No. Interviews were individual and conducted in private rooms. Clinic staff were not present during any interview and had no access to interview content. | Sections 2.3, 2.4 |
| 16 | **Description of sample**  *What are the important characteristics of the sample?* | Table 1 reports participant code, age, sex, marital status, education level, employment status, number of children, and children's HIV status for all twelve participants. Marital status spans single, married, divorced, and widowed; education spans none to tertiary; parity spans nulliparous to six children.  Table 2 additionally reports the timing of HIV diagnosis relative to childbirth for participants with children, together with the basis for each classification.  Age at diagnosis and duration of ART were not systematically collected and their absence is acknowledged as a limitation. | Section 3.1; Tables 1 and 2; Section 6 |
| ***Data collection*** | | | |
| 17 | **Interview guide**  *Were questions, prompts, guides provided by the authors? Was it pilot tested?* | Yes. A semi-structured guide was developed from the research objectives and the literature on fertility among persons living with HIV, with question framing informed by the constructs of the Theory of Planned Behaviour. It was reviewed by the academic supervisor for clarity, coverage, and cultural appropriateness before fieldwork and was piloted with two clinic attendees who were not included in the analysed sample. Piloting led to minor changes in wording and sequencing, chiefly the placement of counseling questions after rapport-building questions. The full guide is provided as Supplementary Data Sheet 2. | Section 2.4; Supplementary Data Sheet 2 |
| 18 | **Repeat interviews**  *Were repeat interviews carried out? If yes, how many?* | No repeat interviews were conducted. Each participant was interviewed once. Eight participants took part in a separate oral member-checking contact after their interview, which validated preliminary interpretations and was not a further data-collection interview. | Sections 2.4, 2.8 |
| 19 | **Audio / visual recording**  *Did the research use audio or visual recording to collect the data?* | Audio recording only, with the permission of each participant. No visual recording was made. Recordings were transcribed verbatim and retained as part of the audit trail. | Sections 2.4, 2.8 |
| 20 | **Field notes**  *Were field notes made during and/or after the interview?* | Yes. Field notes documented contextual observations, non-verbal cues, and immediate analytic impressions. They were triangulated with the transcripts during analysis and were used specifically to record whether an unusually positive assessment of the service had been volunteered by the participant or elicited by a direct question, a distinction that informed how such accounts were weighted. | Sections 2.4, 2.7, 2.8 |
| 21 | **Duration**  *What was the duration of the interviews?* | Interviews lasted between 45 and 90 minutes. Fieldwork ran for three months, from 4 August 2024 to 5 October 2024. | Section 2.4 |
| 22 | **Data saturation**  *Was data saturation discussed?* | Yes, in a dedicated section. Saturation was tracked at two levels following the distinction between code saturation and meaning saturation. Code saturation for counseling-related material was reached by approximately the ninth interview. Meaning saturation required the full set, with later interviews adding nuance rather than replication, most importantly the account of a participant who had received no fertility counseling at all. Interviews eleven and twelve produced no new codes and no new dimensions, and recruitment stopped.  Saturation was assessed across the sample as a whole and not within sub-groups. No claim of saturation is made for men, for any relationship configuration, or for any parity group, and interpretations resting on male accounts are marked as readings of individual accounts at each point where they occur. | Sections 2.6, 3.2, 3.2.2, 4.1, 4.2, 5, 6 |
| 23 | **Transcripts returned**  *Were transcripts returned to participants for comment and/or correction?* | No. Full transcripts were not returned. The manuscript states the reasons, namely the substantial reading burden and, in a study on a stigmatised condition, the disclosure risk created by placing a verbatim transcript in a participant's possession.  In place of transcript return, eight participants were presented orally, in Twi or English as appropriate, with a summary of the situated interpretation of their own account and with the preliminary theme formulation. | Section 2.8 |
| **Domain 3: Analysis and findings** | | | |
| ***Data analysis*** | | | |
| 24 | **Number of data coders**  *How many data coders coded the data?* | Two. PAB conducted the primary coding and interpretation. VLD independently coded a subset of transcripts and reviewed the full coding matrix, the situated accounts, and the theme structure. Both authors met to compare interpretations, and disagreements, which concerned the boundary between two sub-themes within the provider attitudes theme, were resolved by return to the transcripts and reformulation rather than by majority view. The academic supervisor reviewed the analytic framework at two points. | Section 2.5 |
| 25 | **Description of the coding tree**  *Did authors provide a description of the coding tree?* | Yes. Table 3 presents the full structure of three essential themes and nine sub-themes. The initial code list, together with the progression from initial codes through focused codes to sub-themes and themes, is provided as Supplementary Data Sheet 3. Examples of initial code formulations are given in the text, including fear of child infection, cost of child-rearing, and counseling not received. | Section 2.5; Table 3; Supplementary Data Sheet 3 |
| 26 | **Derivation of themes**  *Were themes identified in advance or derived from the data?* | Derived from the data. Codes were generated inductively from participants' own words and no pre-existing code list was imposed. The Theory of Planned Behaviour was not used to structure coding and informed only the later interpretive phase, so the analysis is characterised in the manuscript as inductive with a theoretically informed interpretive phase.  Two sub-themes were reformulated during analysis in response to return to the transcripts, and these reformulations are reported transparently in the text. | Sections 2.5, 3.2.2, 3.2.3 |
| 27 | **Software**  *What software, if applicable, was used to manage the data?* | None. Analysis was conducted manually and no computer-assisted qualitative data analysis software was used. Transcripts were managed in word-processing and spreadsheet files, with coding recorded in margin annotations and consolidated in a coding matrix. The manuscript states the rationale, namely that the small dataset permitted close and repeated engagement with whole transcripts, which suits an interpretive phenomenological approach in which meaning is read within the context of an entire account. | Section 2.5 |
| 28 | **Participant checking**  *Did participants provide feedback on the findings?* | Yes. Member checking was conducted between the eighth week of fieldwork and the four weeks following its close, with eight of the twelve participants. Seven confirmed the interpretations without amendment. One participant qualified the interpretation of her account, indicating that her reservations about childbearing had been shaped more by the absence of a partner than by concern about transmission, which had been the emphasis of the initial reading. The correction was accepted and is reflected in the formulation of the individualized planning sub-theme. No participant proposed changes to the theme structure. | Sections 2.8, 3.2.1 |
| ***Reporting*** | | | |
| 29 | **Quotations presented**  *Were participant quotations presented to illustrate the themes? Was each quotation identified?* | Yes. Tables 4, 5, and 6 present two verbatim quotations for each of the nine sub-themes. Every quotation is identified by anonymised participant code together with sex and marital status, for example P2, Female, Divorced.  The manuscript states explicitly that quotations are drawn from eight of the twelve participants, that all twelve contributed to code and theme development, and that four participants did not describe their counseling encounters in terms that could be quoted meaningfully against the sub-themes. It also states that of the three men in the sample only one is quoted. | Sections 3.2 to 3.2.3; Tables 4, 5 and 6; Section 6 |
| 30 | **Data and findings consistent**  *Was there consistency between the data presented and the findings?* | Yes. Each interpretive passage is linked explicitly to its table and to the named participant accounts it rests upon. Three interpretations that exceeded what the data could support were withdrawn or reformulated during revision and the changes are reported in the text: the inference about PMTCT effectiveness was withdrawn and bounded by Table 2; a sub-theme previously named for perceived judgmental attitudes was renamed once the transcripts showed no participant had described provider judgment; and the specialized services sub-theme was reframed as reported access, with explicit statements that patient accounts cannot establish what the facility provides.  The manuscript records that no service audit was conducted and identifies the alternative explanations the data cannot distinguish between. | Sections 2.2, 3.1 to 3.2.3, 4, 6 |
| 31 | **Clarity of major themes**  *Were major themes clearly presented in the findings?* | Yes. Three essential themes are named in the abstract, summarised in Table 3, and developed in dedicated subsections 3.2.1, 3.2.2, and 3.2.3, each with its own illustrative table and interpretive commentary. Each theme is carried forward into a corresponding subsection of the Discussion. | Sections 3.2 to 3.2.3; Table 3; Section 4 |
| 32 | **Clarity of minor themes**  *Is there a description of diverse cases or discussion of minor themes?* | Yes. Nine sub-themes are reported under the three essential themes and divergent cases are presented rather than smoothed over. These include P1, who had received no fertility counseling at all despite regular attendance and a warm provider relationship; P7 and P9, whose settled reproductive intentions made routinely delivered counseling irrelevant to them; and the member-checking participant whose correction altered the formulation of a sub-theme.  Scope limits are marked where interpretations rest on individual or male accounts, and the coexistence of relational warmth with the absence of counseling content is presented as a deviant case rather than reconciled. | Sections 3.2.1 to 3.2.3; Sections 2.8, 4.1, 4.3, 6 |

**Declaration**

All 32 COREQ items have been considered and addressed in the manuscript or explicitly reported as not undertaken. Items 13, 16, 23, and 27 record procedures that were not carried out, namely the recording of refusals at introduction, the collection of age at diagnosis and ART duration, the return of full transcripts to participants, and the use of qualitative analysis software. In each case the manuscript states the omission and, where relevant, the reason for it and its consequences for interpretation.
