# Supplementary material for: Barriers to comprehensive fertility counseling among persons living with HIV/AIDS in Ghana
Source: Front Reprod Health. 2026 Oct 16;8:1914739. doi: 10.3389/frph.2026.1914739 (PMC13719461; doi:10.3389/frph.2026.1914739)
Supplement: Supplementary file 2 [file Datasheet2.docx]

**INTERVIEW GUIDE**

1. Kindly introduce yourselves
2. When were you diagnosed with HIV?
3. What has your experience been with managing HIV?
4. Are you currently on antiretroviral therapy (ART)?
5. How would you describe your access to and experience with healthcare services?
6. Do you have any children? If so, how many? If yes, are any of your children HIV-positive?
7. Can you describe your current thoughts and feelings about having (more) children?
8. What are your reasons for wanting or not wanting more children?
9. How has your HIV status influenced your thoughts about having children?
10. What factors are most important to you when considering whether to have children?
11. How do healthcare providers address your fertility concerns?
12. Have you received any counselling or support specifically related to fertility and HIV?
13. What challenges or barriers have you faced in pursuing your fertility intentions?
14. How do these challenges affect your decision-making process?
