# Supplementary material for: Barriers to comprehensive fertility counseling among persons living with HIV/AIDS in Ghana
Source: Front Reprod Health. 2026 Oct 16;8:1914739. doi: 10.3389/frph.2026.1914739 (PMC13719461; doi:10.3389/frph.2026.1914739)
Supplement: Supplementary file 3 [file Datasheet3.docx]

**SUPPLEMENTARY DATA SHEET 3**

**De-Identified Qualitative Excerpts and Coded Thematic Summaries**

**Manuscript:** Barriers to Comprehensive Fertility Counseling Among Persons Living with HIV/AIDS in Ghana

**Authors:** Priscilla Asantewaa Boadi, Victor Luckyboy Dzramado

**Journal:** Frontiers in Reproductive Health, HIV and STIs specialty section. Article type: Original Research.

***Note on this version.*** *This file replaces the version uploaded at submission. It has been revised so that the sub-theme names, the participant attributions and the analytic wording correspond exactly to the published article. Three changes were made. Sub-theme 2.2 is now named "Counseling misaligned with settled reproductive intentions", replacing the earlier "Perceived judgmental attitudes", because no participant described a provider behaving, speaking or appearing judgmental. Sub-theme 3.3 is now named "Reported access to specialized reproductive services", replacing the earlier "Availability of specialized reproductive services", because patient accounts cannot establish what the facility provides. Participant attributions have been corrected against the source transcripts and now match Tables 4 to 6 of the article.*

***Note on de-identification.*** *All identifying information has been removed. Participants are referenced by anonymised codes (P1 to P12) accompanied only by broad, non-identifying descriptors. All participants were living with HIV and receiving antiretroviral therapy, consistent with the eligibility criteria. Excerpts are verbatim except for minor clarifying punctuation.*

**PART A. De-Identified Qualitative Excerpts**

**Theme 1: Quality of Reproductive Health Counseling and Education**

**Sub-theme 1.1: Comprehensive PMTCT education**

*“That having the disease doesn't deter one from not having kids and in our condition we can still have healthy babies. Yes please, every time I come for checkup. I am just hopeful that when the time comes for me to have a baby I can also go through my due months and have a healthy baby as any other woman out there.”*

(P8, female, single)

*“That even if I want to have a child again, taking my medications will not affect the baby. I don't want to infect any child I might have.”*

(P9, female, married)

**Sub-theme 1.2: Safer conception counseling for serodifferent couples**

*“There are drugs I can take to prevent my baby from having the disease and how I can get pregnant with my partner without infecting him as well. Yes please, the doctors and nurses have been very supportive. I am counselled each time I visit the hospital.”*

(P2, female, divorced)

*“That I can still give birth in my condition without affecting my child. I am been pressured by my husband and also I have a boy so I want a baby girl too.”*

(P6, female, married)

**Sub-theme 1.3: Individualized reproductive health planning**

*“Not yet, but I think along the line I will receive counselling on fertility issues. They give me drugs to that effect. I really wish to have children but I am also thinking about whether my children will contract the sickness. Also because of this I intend not to have children.”*

(P1, female, single)

*“We are being taught every time I visit the hospital and I will rate them about 70%. I want to have more babies but I don't have a partner at the moment.”*

(P2, female, divorced)

**Theme 2: Healthcare Provider Attitudes and Support**

**Sub-theme 2.1: Supportive and encouraging provider approaches**

*“I have a very cordial experience with the health workers, I am saying so because they granted me unlimited access to have time with them and also have time for me.”*

(P1, female, single)

*“They are friendly, confidential and patient with me.”*

(P6, female, married)

**Sub-theme 2.2: Counseling misaligned with settled reproductive intentions**

*“Yes but I don't pay much attention because I have enough kids. I was not really worried because I have enough kids. I don't want children again.”*

(P7, male, married)

*“Yes we are taught but I don't need babies again so I don't really pay attention to it.”*

(P9, female, married)

**Sub-theme 2.3: Provider comfort with fertility discussions**

*“Yes please, every time I come for checkup.”*

(P8, female, single)

*“Yes they do and we are been encouraged to have children and live our lives as any other person. It has change my perspective on marriage and child bearing.”*

(P3, female, married)

**Theme 3: Access to Comprehensive Reproductive Health Services**

**Sub-theme 3.1: ART access and adherence support**

*“I have access and by taking the medications given I feel healthy.”*

(P8, female, single)

*“I receive all the necessary services and I have easy access to all my health care needs.”*

(P3, female, married)

**Sub-theme 3.2: Integration of reproductive health into HIV care**

*“We are being taught every time I visit the hospital and I will rate them about 70%. I am counselled each time I visit the hospital.”*

(P2, female, divorced)

*“Yes please, we are taught at the hospital after child bearing in our condition and our general wellbeing.”*

(P3, female, married)

**Sub-theme 3.3: Reported access to specialized reproductive services**

*“I want to have more babies but I don't have a partner at the moment. I am hesitant in child bearing but hopefully one day I will follow all the processes so I can get a healthy baby.”*

(P2, female, divorced)

*“I want a baby but doesn't want to put him/her at risk because of the disease.”*

(P6, female, married)

**PART B. Coded Thematic Summaries**

The tables below display the analytic progression for each theme, moving from initial descriptive codes that stayed close to participants' language, through focused interpretive codes, to the sub-themes and themes reported in the article. The final column names the participants whose accounts illustrate each sub-theme in the published tables. This material documents the transparency and dependability of the analysis.

**Table B1. Coded thematic summary for Theme 1: Quality of Reproductive Health Counseling and Education**

| **Initial codes** | **Focused code** | **Sub-theme** | **Participants illustrating** |
| --- | --- | --- | --- |
| Healthy babies possible with HIV; medication will not affect the baby; taught at every checkup | PMTCT knowledge restores reproductive possibility | 1.1 Comprehensive PMTCT education | P8, P9 |
| Drugs to protect the baby; conceiving without infecting the partner; giving birth without affecting the child | Counseling that reaches the partner as well as the child | 1.2 Safer conception counseling for serodifferent couples | P2, P6 |
| Counseling not yet received; drugs given in place of counseling; taught at every visit; no partner at present; service rated about 70% | Counseling not directed to the individual's circumstances, or not received at all | 1.3 Individualized reproductive health planning | P1, P2 |

**Table B2. Coded thematic summary for Theme 2: Healthcare Provider Attitudes and Support**

| **Initial codes** | **Focused code** | **Sub-theme** | **Participants illustrating** |
| --- | --- | --- | --- |
| Cordial experience; unlimited access and time; friendly, confidential and patient | Relational warmth, which did not by itself deliver counseling content | 2.1 Supportive and encouraging provider approaches | P1, P6 |
| Not paying attention because of having enough children; does not need babies again | Participants' own withdrawal of attention from counseling that did not match their settled intentions | 2.2 Counseling misaligned with settled reproductive intentions | P7, P9 |
| Counselled at every checkup; encouraged to have children and to live as any other person | Providers raising fertility without waiting to be asked | 2.3 Provider comfort with fertility discussions | P3, P8 |

**Table B3. Coded thematic summary for Theme 3: Access to Comprehensive Reproductive Health Services**

| **Initial codes** | **Focused code** | **Sub-theme** | **Participants illustrating** |
| --- | --- | --- | --- |
| Access to ART; feeling healthy on medication; easy access to all health care needs | ART access as the precondition for entertaining childbearing | 3.1 ART access and adherence support | P3, P8 |
| Counselled at every visit; reproductive teaching located within general wellbeing education | Reproductive health built into the routine HIV visit | 3.2 Integration of reproductive health into HIV care | P2, P3 |
| No partner at present; hesitant but hopeful of following all the processes one day; wanting a baby but unable to resolve the risk to the child | No referral, specialist consultation or structured reproductive plan reported by these participants | 3.3 Reported access to specialized reproductive services | P2, P6 |

**Table B4. Participants whose accounts illustrate each sub-theme in the published article**

| **Theme** | **Sub-theme** | **Participants illustrating** |
| --- | --- | --- |
| Quality of counseling and education | 1.1 Comprehensive PMTCT education | P8, P9 |
|  | 1.2 Safer conception counseling for serodifferent couples | P2, P6 |
|  | 1.3 Individualized reproductive health planning | P1, P2 |
| Provider attitudes and support | 2.1 Supportive and encouraging provider approaches | P1, P6 |
|  | 2.2 Counseling misaligned with settled reproductive intentions | P7, P9 |
|  | 2.3 Provider comfort with fertility discussions | P3, P8 |
| Access to reproductive services | 3.1 ART access and adherence support | P3, P8 |
|  | 3.2 Integration of reproductive health into HIV care | P2, P3 |
|  | 3.3 Reported access to specialized reproductive services | P2, P6 |

Note. The final column names the participants whose accounts are quoted for each sub-theme in Tables 4 to 6 of the article. It records illustration, not frequency of expression, and no numerical claim is made from it. All twelve participants contributed to code development and to the construction of the theme structure. Four participants discussed diagnosis, treatment and childbearing intentions but did not describe their counseling encounters in terms that could be quoted meaningfully against these sub-themes, and no attempt was made to press their accounts into illustrative service. Of the three men in the sample, one is quoted, and readings resting on his account are marked as such in the article and are not generalized to men attending the clinic.
